# Resistance Sources to Brown Blotch Disease (*Pseudomonas tolaasii*) in a Diverse Collection of *Pleurotus* Mushroom Strains

**DOI:** 10.3390/pathogens8040227

**Published:** 2019-11-09

**Authors:** Benjamin Azu Okorley, Frederick Leo Sossah, Dan Dai, Shuai Xu, Zhenghui Liu, Bing Song, Hongyan Sheng, Yongping Fu, Yu Li

**Affiliations:** 1 International Cooperation Research Center of China for New Germplasm and Breeding of Edible Mushrooms, Jilin Agricultural University, Changchun 130118, China; 2 Department of Plant Pathology, Jilin Agricultural University, Changchun 130118, China; 3 Department of Plant Pathology, Washington State University, Pullman, WA 99164-6430, USA

**Keywords:** brown blotch disease, *Pseudomonas tolaasii*, *P. pulmonarius*, *P.* cf. *floridanus*, *P. ostreatus*, mushroom, IMP, IMSS and resistance

## Abstract

Brown blotch disease (BBD) caused by *Pseudomonas tolaasii* is one of the most devastating diseases of *Pleurotus* spp. worldwide. Breeding for resistant strains is the most effective method for controlling BBD. To identify resistant germplasm for BBD management, 97 strains comprising 21 *P.* cf. *floridanus*, 20 *P. ostreatus,* and 56 *P. pulmonarius* were screened by two different methods; namely, inoculation of the pathogen on the mushroom pileus (IMP) and on the spawned substrate (IMSS) under controlled conditions. Out of the 97 strains screened, 22 *P. pulmonarius,* and four *P.* cf. *floridanus* were moderately resistant to BBD using the IMP method. Eleven *P. pulmonarius,* six *P*. cf. *florida,* and one *P. ostreatus* strains were highly resistant to BBD using the IMSS method. All of the 97 strains showed varying degrees of susceptibility using the IMP method, but eight strains were completely resistant using the IMSS method. Combining these two methods, five strains were highly resistant (four *P. pulmonarius* and one *P.* cf. *floridanus*) and 11 were moderately resistant (eight *P. pulmonarius* and three *P.* cf. *floridanus*). The resistance sources to *P. tolaasii* identified in *P. pulmonarius* and *P.* cf. *floridanus* could be used for further breeding of *Pleurotus* spp.

## 1. Introduction

The *Pleurotus* genus consists of approximately 50 recognized species, including more than 10 important species that are highly nutritious and among the most commercially cultivated edible fungi in the world [1,2]. In China, *Pleurotus ostreatus* (oyster mushroom), *Pleurotus pulmonarius* (phoenix mushroom), and *Pleurotus* cf. *floridanus* (Florida oyster mushroom) are among the most commonly cultivated species with considerable economic value. Their production exceeds 500 million tons annually for both domestic consumption and export purposes [3]. 

However, one of the important diseases that threatens *Pleurotus* mushroom production is brown blotch disease (BBD), caused by *Pseudomonas tolaasii*. The bacterium can also infect other economically important mushrooms, such as *Agaricus bisporus, Lentinus edodes,* and *Flammulina velutipes* [4,5,6,7]. *P. tolaasii* can infect all the stages of *Pleurotus* spp. fruiting body [8] by secreting tolaasin, a core toxin used to cause BBD on the mushroom [9]. Tolaasin causes lysis of hyphal cell membranes and induces an increase in the amount of active tyrosinase, leading to melanin synthesis at the site of infection [10]. The characteristic symptoms of BBD include initially small, separated pale brown discolorations, which then spread all over the pileus surface. When BBD is severe, blotches become darker and sunken, ultimately rendering the pileus unappealing for human consumption [11]. These symptoms lead to reduced quality [12,13], which can cause a significant economic loss of up to 25% [14,15]. 

Over the past years, strategies to control BBD and interrupt pathogen spread have included the use of chemicals (antibiotics, hydrogen peroxide, and chlorinated compounds), management of host environment, and biocontrol agents (plant extracts and antagonistic microbes) [16,17,18,19,20,21,22]; however, none of these strategies appear to be fully efficient in managing the disease. There is even an increased concern about the effects of these chemicals on consumers. Further, the ability of *P. tolaasii* to tolerate adverse environmental conditions through phenotypic plasticity makes BBD control difficult [23,24,25], thereby resulting in huge crop losses during severe epidemics. 

At present, the use of resistant strains is the most efficient and long-term method for BBD control [26,27,28], since it not only reduces the loss of the mushroom quality, but also the cost associated with measures undertaken to control the disease. Therefore, resistance sources in *Pleurotus* germplasm are needed for the sustainable management of BBD. Several studies have described partial resistance to BBD in *A. bisporus* strains [26,27,29,30]. However, only one has reported partial resistance of *P. ostreatus* strains to *P*. *tolaasii* [31]. 

The *Pleurotus* genus contains many species with genetically diverse strains in the wild, and many are also maintained by various mycological germplasm repositories [32,33]. Additionally, interspecific hybridization can occur between species [34]. Therefore, developing BBD-resistant strains may rely on characterization and incorporation of resistance genes contributed from wild strains or closely related species. Despite the large collection of *Pleurotus* species in many germplasm repositories, little is known about their resistance to BBD. Thus, searching for novel sources of resistance to BBD is of high priority. 

In screening for disease resistance in mushrooms, several studies have recommended the artificial inoculation of the pathogen and assessment of the host response under a controlled environment. Oliver et al. [27] described the use of the direct inoculation of the pathogen on the mushroom pileus/cap (IMP) method to screen for BBD resistance in *A*. *bisporus*. However, Zhang et al. [31] could not clearly distinguish between the host responses from *P*. *ostreatus* and proposed the inoculation of the pathogen on the mushroom spawned substrate (IMSS) as the effective method for successful evaluation of BBD resistance in *P*. *ostreatus*. The objectives of this study were to (1) evaluate the efficacy of the IMP screening method on *Pleurotus* species; (2) determine the influence of inoculum dose, temperature and disease assessment time on the resistance test; and (3) identify possible resistance sources from a collection of 97 *Pleurotu*s strains.

## 2. Results

### 2.1. Pathogenicity of P. tolaasii Strain Pt011W

Morphological and molecular analyses were used to confirm the identity of bacterial strain Pt011W in this study. Results from BLASTn alignments of the 16S rRNA, rpoβ, and tolaasin biosynthesis gene sequences showed 98–100% homology to the known sequences of *P. tolaasii* strains deposited in GenBank (MN630174, MN630175, and MN630176, respectively). A neighbor-joining phylogenetic tree confirmed strain Pt011W as *P. tolaasii* with 100% branch support based on the concatenated 16S rRNA and rpoβ sequence data of Pt011W and other published sequences of *Pseudomonas* spp. (Supplementary Figure S1). 

**Figure 1:**
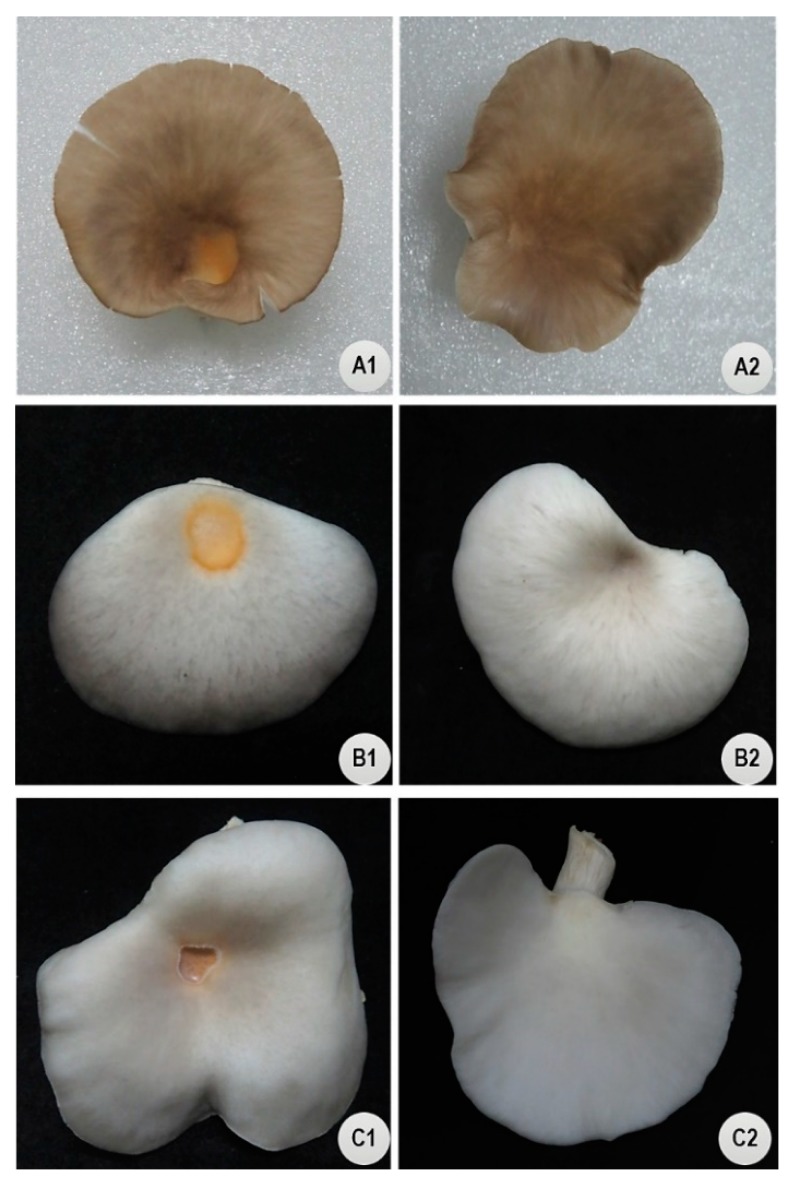
Brown blotch disease (BBD) symptoms caused by *P. tolaasii* strain Pt011W on fruiting bodies of three *Pleurotus* strains, 48 hours after inoculation. (**A1–C1**) Botch symptoms on mushroom caps inoculated with ≈ 2.6 × 10^6^ CFU/20 µL of *P. tolaasii*. (**A2–C2)** Healthy mushroom inoculated with sterile water. (**A1**,**A2**) *P. pulmonarius* (PPU), (**B1**,**B2**) *P.* cf. *florida* (P282), (**C1**,**C2**) *P. ostreatus* (P0).

Artificial inoculation of *P. tolaasii* Pt011W on the mushroom pileus surface induced typical brown blotch symptoms on strain P0 (*P. ostreatus*), P282 (*P.* cf. *floridanus*), and PPU (*P. pulmonarius*) 24 hours after inoculation (HAI). The symptoms were yellow, brown, slight to extensively sunken, and were localized to the portion of the cap on which inoculum was applied (Figure 1). Caps treated with sterile water as controls remained asymptomatic. Re-isolation of the causal agent consistently yielded the same pathogen (*P. tolaasii*) to confirm Koch’s postulates. 

### 2.2. Efficacy of the IMP Method for Resistance Evaluation.

**Figure 2:**
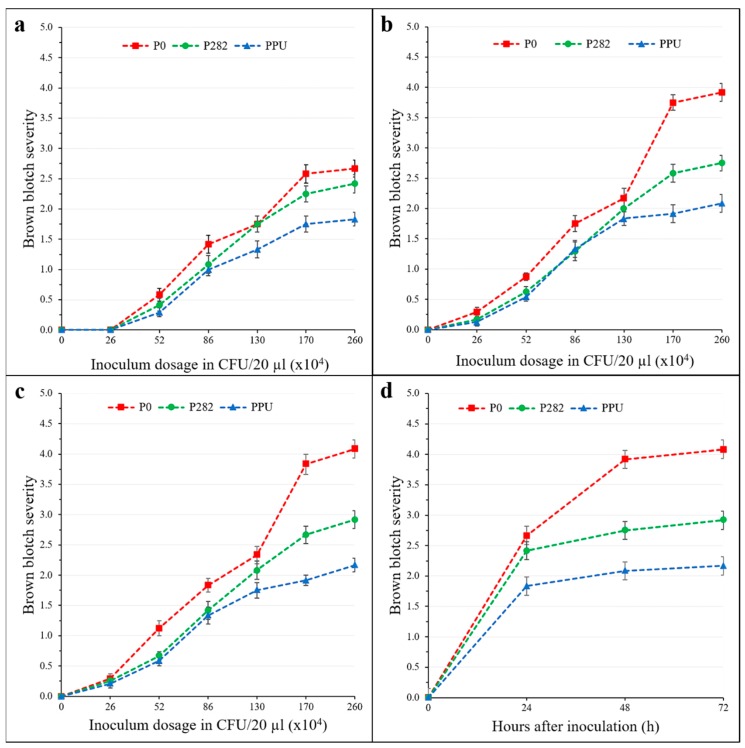
Development of BBD over an infection time course from 24–72 HAI with *P. tolaasii* strain Pt011W inoculum suspension on mushroom caps of three *Pleurotus* strains (P0, P282, and PPU) in a controlled environment. (**a**–**c**) Blotch symptom severity with increasing levels of inoculum dosage at 24, 48, and 72 HAI, respectively. (**d**) Symptom progression on the three strains using the optimum inoculum dosage. Disease severity (DS) rating was based on a 0–5 scale according to the extent of tissue discoloration and nature, where 0 = no symptom; 0.5 = very pale lesion difficult to distinguish from the cap; 1 = level yellowish lesion; 2 = level brown lesions; 3 = slightly sunken lesion; 4 = extensive brown sunken lesion; 5 = extensive dark sunken lesions occasionally with sticky cytosolic material [28]. Data are mean disease severity scores of two trials (at 18 °C and 21 °C), each with six bags treated (three fruiting caps inoculated/bag)/strain. Bars are the standard errors of the means.

Evaluation of BBD symptom severity using a 0–5 scale ensured the consistent discrimination of strains regarding resistance to *P. tolaasii* Pt011W (Table 1). The 3-way interactive effects of strains (S), temperatures (T), and inoculum dosages (D) (S × T × D), and all other effects involving T did not significantly affect disease severity (DS) for the length of HAI. In other words, though different incubation temperatures (21 °C and 18 °C) were used to induce the fruiting of different species it did not have an effect on DS. In contrast, DS was significantly affected by the interaction of S × D, 24 to 72 HAI. Examination of the main effects showed increasing inoculum dosage corresponded with severe blotch symptoms, regardless of assessment time (Figure 2a–c). At 24 HAI, the overall average DS of the three strains treated with 1.3 × 10^7^ CFU/mL inoculum suspension (providing ≈2.6 × 10^5^ CFU/20 µL on the cap surface) was not significantly different from the control, except at 48 HAI, where it was difficult to distinguish the symptoms on the cap visually (Figure 2a–c). The inoculation on mushroom caps using 20 µL of 1.3 × 10^8^ CFU/mL inoculum suspension gave the most severe symptoms for the three strains (Figure 2d). In order to prevent the escape of strains from infection during the resistance test, a high dosage of the inoculum was required. Therefore, the 1.3 × 10^8^ CFU/mL inoculum suspension, providing ≈ 2.6 × 10^6^ CFU/20 µL on the cap surface, was selected as the optimum inoculum dosage for infection and was used in the subsequent trial.

### 2.3. Determination of the Best Time for Disease Assessment 

The BBD progression curves for the three strains inoculated with the optimum inoculum dosage are shown in Figure 2d. BBD severity differed significantly (*p <* 0.05) among the strains as the infection progressed from 24 to 48 and to 72 HAI (Table 1). Strain P0 recorded the highest DS compared to P282 and PPU. Similarly, the variance component attributed to strain increased from 2.44 to 4.74%, indicating the difference between the strains as the period of incubation increased. However, the magnitude of increase in DS slowed down from 48 to 72 HAI. Hence, the most meaningful time point to carry out disease assessment with low variability between replicates (error = 8.47%) was 48 HAI. 

At this time point, and with the selected optimum inoculum, the mean DS scores of the strains under the two different incubation environments (at 21 °C and 18 °C) were reproducible, since the interactions between temperature and all other terms in the model were absent. The disease response of the strains between the two environments was positively correlated (*r =* 0.97, *p* < 0.05). 

### 2.4. Screening for Resistance to BBD Based on the IMP Method

In 2017, all 97 strains (56 *P. pulmonarius,* 21 *P.* cf. *floridanus,* and 20 *P. ostreatus*) examined by the IMP method produced fruiting bodies with varied cap colors (Table 2, Table 3 and Table 4). After 48 hours of inoculation, blotch symptoms appeared on the fruiting bodies of all the strains, but varied in lesion morphology. The average DS1 ± standard deviation was 1.95 ± 0.70, 2.23 ± 0.61, and 2.86 ± 0.77 for *P. pulmonarius* (Table 2)*, P.* cf. *floridanus* (Table 3), and *P. ostreatus* (Table 4), respectively. None of the strains were immune or completely resistant to *P. tolaasii;* however, significant (*p* < 0.05) variations in DS among the strains in each species group were obtained. Based on the average DS, the strains were rated as highly resistant (HR) (DS1 ≤ 0.5), moderately resistant (MR) (DS1 ≤ 1.5), moderately susceptible (MS) (DS1 ≤ 2.5), susceptible (S) (DS1 ≤ 3.5), or highly susceptible (HS) (DS1 > 3.5) (Table 2, Table 3 and Table 4). 

None of the 97 strains were rated as HR. Twenty-two *P. pulmonarius* and four *P.* cf. *floridanus* strains (P290, P287, P301, and P328) were MR (Table 2 and Table 3). None of the 20 *P. ostreatus* strains were HR or MR. Four *P. ostreatus* strains (P173, P40, P0, and P167) were rated HS. Mushroom cap colors showed no relationship with the varying susceptibility levels among the strains. For example, the brown-colored *P. pulmonarius* strains recorded both low and high DS scores (Table 2). 

### 2.5. Screening for Resistance to BBD Based on the IMSS Method 

**Figure 3:**
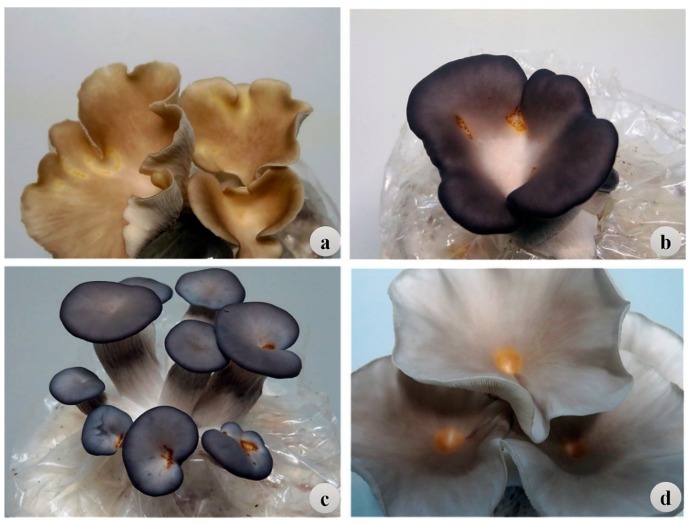
Typical symptoms of BBD on mushroom fruiting bodies after inoculation of *P. tolaasii* on the mushroom spawned substrate (**a–c**) and on the mushroom fruiting cap (**d**). (**a**) *P. pulmonarius*, (**b**) *P.* cf. *florida*, (**c**,**d**) *P. ostreatus.*

To mimic the primary route of infection in mushroom farms, the same 97 strains were assessed by artificial inoculation of the pathogen on the mushroom spawned substrate (IMSS) in the succeeding trial in 2018. Throughout the fruiting period, the blotch lesions were observed on the primordial and open pileus fruiting bodies (Figure 3). The blotch lesion number and size increased with growth and expansion of mushroom fruiting bodies. Similar symptoms were observed on the second flush of fruiting bodies. The average DS2 ± standard deviation was 1.18 ± 0.76, 1.23 ± 0.95, and 1.92 ± 0.77 for *P. pulmonarius* (Table 2)*, P.* cf. *floridanus* (Table 3), and *P. ostreatus* (Table 4), respectively. The mean DS2 varied significantly (*p* < 0.05) among the strains in each species group. Based on the average DS from the two flushes, the strains were rated as HR (DS2 ≤ 0.5), MR (DS2 ≤ 1.0), MS (DS2 ≤ 1.5), S (DS2 ≤ 2.0), or HS (DS2 > 2.0) (Table 2, Table 3 and Table 4).

Fifty-eight strains had many blotches covering >1% of cap surface (DS2 > 1) and were rated MS, S, or HS (Table 2, Table 3 and Table 4). In contrast, 26 strains of *P. pulmonarius* (Table 2), 10 of *P.* cf. *floridanus* (Table 3), and three of *P. ostreatus* (P346, P317, P176) (Table 4) showed few spotty blotches covering <1% of the mushroom cap surface area and were rated either HR or MR. Among the 18 HR strains (11 *P. pulmonarius,* six *P.* cf. *floridanus,* and one *P. ostreatus*), three of *P. pulmonarius* (F122, F324, F334), four of *P.* cf. *floridanus* (P287, P289, P334, P335), and one of *P. ostreatus* (P346) consistently showed asymptomatic fruiting bodies in the two flushes of mushroom fruiting (Table 2, Table 3 and Table 4, respectively). Their responses suggest complete resistance to BBD. However, previous screening by the direct IMP method revealed the susceptibility of their caps to *P. tolaasii* Pt011W. For instance, P346 of *P. ostreatus* and P334 of *P.* cf. *floridanus* recorded high DS1 > 2.5 on the IMP disease rating scale.

### 2.6. Comparison of Variation in Susceptibility to BBD due to Species and Strains 

In both screening methods, the nested method (ANOVA) applied for strains within species revealed significant differences in BBD severity among the species groups (Table 5). However, a large broad-sense genetic variation denotes that level in the *Pleurotus* taxa is of interest for further research and optimal resource allocation. The variance component analysis revealed that little variation in disease response occurred between species (25.4% and 14.96% when using the IMP and IMSS, respectively), and also between replicates within strains. Most of the detected variations in disease response occurred between strains (60.76% and 72.35% when using the IMP and IMSS, respectively), which indicates that regardless of the method used, the individual strains in the *Pleurotus* taxa determined resistance to *P. tolaasii,* even more than species.

### 2.7. Pleurotus Strains with Less Susceptibility to BBD Using Both IMP and IMSS Methods

Based on the IMP DS scale, 26 of the 97 strains were classified MR (DSI ≤ 1.5). With the IMSS DS scale, 18 and 21 strains were classified HR and MR, respectively (DS2 ≤ 1). We also found some strains with lower DS ratings on both the IMP and IMSS method DS scales. These strains showed less intense brown discolorations and had few spotty blotches affecting <1% of the mushroom cap surface area. For the strains which showed complete resistance to *P. tolaasii* in the IMSS method, an overall disease severity index (DI) integrated the disease severity scales of both the IMP and IMSS methods by a simple formula required to give an accurate measure of their resistance. Further, the formula was applied to all strains to enhance the selection of resistant strains and to pyramid different resistance mechanisms against *P. tolaasii*. Based on the DI of each strain, 16 strains (12 *P. pulmonarius* and four *P.* cf. *floridanus*) had less susceptibility on the integrated scale (DI ≤ 25%) (Table 6) (details are reported in Table 2, Table 3 and Table 4). Among them, *P. pulmonarius* F122, F324, F344, F9, and *P.* cf. *floridanus* P287 were HR (DI ≤ 15%), while the remaining 11 strains were MR (DI = 15–25%). The remaining 81 strains, including all the 20 strains of *P. ostreatus,* were in the MS, S, or HS class.

We further found a significant positive correlation between the two screening methods for strains of *P. pulmonarius* (*r =* 0.28 and *p* < 0.05), yet weakly correlated magnitude and rank changes in disease response were observed. In contrast, there was no linear relationship for strains of *P.* cf. *floridanus* (*r =* 0.24 and *p* > 0.05) and *P. ostreatus* (*r =* 0.01 and *p >* 0.05) between the two methods. 

### 2.8. Bacterial Detection and Numbers in Substrate Bags 

**Figure 4:**
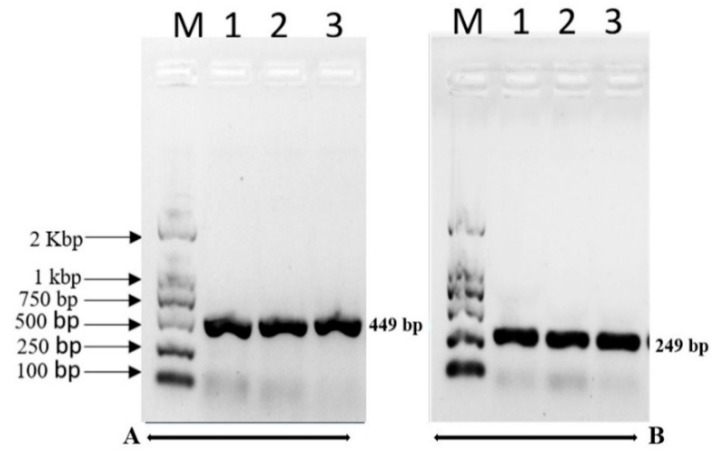
Molecular detection of *P. tolaasii* using DNA template isolated from three selected colonies (Lanes 1, 2, and 3) growing on PAF agar. Amplified products were obtained in a nested PCR assay. Profile A is the first-round PCR product (449 bp) with primer set Pt-1A/Pt-1D1, and B second-round PCR product (249 bp) with the internal primer set Pt-PM/Pt-QM as described in Lee et al. [36]. They were separated in agarose gel electrophoresis. Lane M, Trans2K DNA Marker (TransGen Ltd., China). DNA fragments of 449 bp and 249 bp indicate the presence of *P. tolaasii*.

After the first flush harvest, *P. tolaasii* was re-isolated from 10 g of the mycelia colonized substrates onto *Pseudomonas* Agar for detection of fluorescein (PAF agar). The bacteria colonies grew after incubation at 25 °C for 48 h. Their cultural characteristics were consistent with previous descriptions of *P. tolaasii* [35]. Based on PCR amplification of the tolaasin biosynthesis gene, these colonies were confirmed as *P. tolaasii* (Figure 4). The isolation and recovery of the bacterium from brown blotch symptomatic caps and from spawned substrates in the experiments show *P. tolaasii* was the pathogenic agent causing BBD on the mushroom fruiting bodies. The population of *P. tolaasii* recovered from the spawn substrate varied significantly among the sampled strains of *P. pulmonarius* and of *P.* cf. *floridanus,* with an exception for those of *P. ostreatus* (Table 7). Strains rated as resistant (F359, P176, P334) in the IMSS method showed low population levels of *P. tolaasii*.

## 3. Discussion

*Pleurotus* species are among the important cultivated mushrooms that are susceptible to BBD. To help minimize economic losses in commercial production of *Pleurotus* mushroom, the use of resistant strains is considered the most effective and desirable solution for BBD management. Among *Pleurotus* species, only *P*. *ostreatus* strains have been evaluated for BBD resistance [31]. Therefore, it is imperative to find and develop resistant strains of other *Pleurotus* species to control BBD. In this study, we screened 97 strains from three major cultivated *Pleurotus* species (*P*. *pulmonarius*, *P.* cf. *floridanus,* and *P*. *ostreatus*) to identify germplasm resistant to BBD using the IMP and IMSS methods.

We first found the IMP screening method could be used to assess the response of the three species in the *Pleurotus* genus to the pathogen *P*. *tolaasii*, which is consistent with previous studies that used this method on other kinds of mushrooms [27,29,37,38,39]. However, Zhang et al. [31] reported that it was difficult using the IMP method to distinguish between the host response or symptoms in *P*. *ostreatus*. This might be due to the selected disease scale and the length of the disease incubation period used for symptom evaluation. The main advantage of the IMP method is the application of a uniform inoculum directly on the host pileus for resistance evaluation. The high inoculum concentration (1 × 10^8^ CFU/mL) applied on the pileus was a complete resistance detection assay [40], which minimized the chance of disease escape to make immunity detection easy. Also, the IMP depicted all sources of inoculum that may land on the mushroom pileus, especially secondary infections that occur during the cultivation of the mushroom in farms, transportation, storage, and marketing [12]. 

The three *Pleurotus* species had a different incubating temperature for fruiting. However, it was not surprising to find that temperature had no significant influence on BBD severity. This was consistent with Wong and Preece [41], who noted changes in temperature and relative humidity during cultivation had no effects on BBD severity in *A. bisporus*. Thus, it was possible to compare and select resistant strains under the two different incubation temperatures.

Based on the IMP method, all of the 97 *Pleurotus* strains were susceptible to *P*. *tolaasii* infection, but the degrees of susceptibility varied among the strains. For example, among the *P. pulmonarius* strains, F65, F365, and F18 had significantly different means for disease severity, ranging from 1.07–3.30 (Table 2). Oliver et al. [27] also obtained a wide variation in response to BBD among *Agaricus* strains. The variations in susceptibility among the strains may be attributed to differences in genotype [33], fruiting body nutrient composition, texture, and quality [10,42]. Murata [43] noticed the activity of tolaasin, the core virulence factor, was triggered by the host and the signal strength may be influenced by the chemical composition of the mushroom fruiting body. Estrada et al. [37] also found differences in blotch symptom severity between two strains of *P. eryngii*, which contained varied inherent levels of copper that is a cofactor for tyrosinase in melanin synthesis or the browning of the diseased portions on the pileus [44,45]. 

Compared to the IMP method, the IMSS method revealed a marked difference in resistance levels among the 97 strains. For example, among the *P.* cf. *floridanus* strains, P282 was highly susceptible, while P287 was highly resistant. Moreover, a higher level of resistance to BBD was observed for some strains as their fruiting bodies were completely symptomless. This contrasts with Zhang et al. [31], who observed partial resistance in 37 strains of *P. ostreatus* using the IMSS method. The study showed that besides the host pileus interacting with *P. tolaasii,* host vegetative mycelia interaction with *P. tolaasii* could prevent pathogen spread onto the pileus surface during the initial stages of fruiting body development. The vegetative mycelia of *Pleurotus* spp. are known to degrade living bacteria [46], and the antagonistic interactions between the host mycelia and the pathogen may reduce *P. tolaasii* population in the spawned substrate, which leads to less pathogen load reaching the mushroom pileus. The *Pleurotus* strains showed variable levels of *P. tolaasii* (CFU) in the substrate after the first flush harvest. Thus, the resistance of the strains may be related to *P. tolaasii* population in the spawn substrate. In contrast, Zhang et al. [31] reported no relationship between mushroom resistance and *P. tolaasii* population in the substrate.

Further, a possible resistance strategy obtained with the IMSS method is the escape of the host’s fruiting body from the minimum inoculum dosage required for successful infection. We found that three strains during the preliminary evaluation of the IMP method could carry ≈2.6 × 10^5^ cells of *P. tolaasii*/cap without showing symptoms on their fruiting bodies. Similar observations had been made on *Agaricus* mushrooms [27,47].

The classification of the strains into the susceptible and resistant groups was influenced by the methods (IMSS or IMP) used for assessment. For example, some strains (F324, P334, and P346) were completely symptomless using the IMSS; however, they showed symptoms in the IMP method. Additionally, there was a weak or no linear relationship for the responses of strain between the two screening methods. The discrepancy between the methods may be due to differences in the disease scales used for the study. Further, because the specific disease resistance mechanisms at the different mushroom growth stages (vegetative mycelia and fruiting) are poorly understood, it is possible that the IMP and IMSS screening methods exhibit distinct disease resistance mechanisms. The tendency of hosts to advance defenses throughout their developmental stages has been reported in numerous plant pathological systems and are considered paramount in disease management [48,49,50]. Considering the sources of pathogen inoculum and dissemination in mushroom farms, as well as the host developmental stage at which infection occurs, to identify resistance to BBD in a collection, the selection of genotypes was based on those with lowered DS in both screening methods, using their overall disease index (DI). The results suggest that the data from IMP and IMSS screening methods should be combined to enhance the selection process when evaluating resistance to BBD, because they reflect the primary and secondary routes of infection by *P*. *tolaasii* and demonstrate the accurate total resistance in the mushroom. 

Our study identified *P*. *pulmonarius* and *P.* cf. *floridanus* strains as sources of resistance to *P. tolaasii.* On average, *P. pulmonarius* exhibited higher levels of resistance to *P*. *tolaasii* than *P.* cf. *floridanus* and *P*. *ostreatus*. Sixteen strains identified herein as partially resistant (both HR and MR) have the potential to reduce mushroom production losses due to BBD under both primary and secondary infections. Five strains, F122, F324, F344, and F9 of *P. pulmonarius* and P287 of *P.* cf. *floridanus,* ranked highly resistant. Compared to the other two species, none of the *P. ostreatus* strains included in the study were classified as partially resistant based on the DI. However, the large variance in susceptibility attributed to strains in the study indicated that individual strains rather than species are the most needed for breeding against *P. tolaasii* in further study. The *Pleurotus* collection originated from five provinces in China and two other countries (Pakistan and USA). However, there were no obvious trends for the association of BBD resistance to biogeography. Also, there was a range of resistance within the collection for both wild and cultivated strains. For example, most of the *P. pulmonarius* strains that were resistant to *P*. *tolaasii* were wild-type strains. In order to exploit the five highly resistant *Pleurotus* strains, exhaustive histological and molecular work is needed to elucidate the precise resistance mechanisms present in the *Pleurotus* collection. Further analyses on the nutritional composition and structural characteristics of the resistant strains are required in order to introgress the resistance to other popular strains by breeding. 

## 4. Materials and Methods 

### 4.1. Mushroom Strains and Cultivation Under Controlled Environment

For the germplasm evaluation, a total of 97 strains of *Pleurotus* (56 *P. pulmonarius,* 20 *P. ostreatus,* 21 *P.* cf. *floridanus*) were identified and provided by the Engineering Research Center for Edible and Medicinal Fungi (ERCEMF), Jilin Agricultural University, Jilin Province, China (Supplementary Table S1). These strains were cultured on potato dextrose agar (PDA) and incubated at 25 °C for 5–8 days to resume active growth. Two different trials were performed by different inoculation methods: The first trial was inoculated using the IMP method from August to November 2017, and the second trial was inoculated using the IMSS method from April to July 2018. Both experiments were carried out in controlled incubation rooms at the Mushroom Base of Jilin Agriculture University, China. The cultivation substrate filled into 9 × 8 × 20 cm polyethylene bag consisted of 2:1:2 ratio (w/w) of 40% sawdust, 19% wheat straw, 40% corn straw, supplemented with 1% (w/w) calcium carbonate, and moistened to 60%. The bags were autoclaved at 121 °C for 2 hours (h) and inoculated with fungal mycelia (10 bags/strain). For each trial, 1555 and 300 inoculated bags were completely randomized, respectively, in two incubation rooms at 25 ± 1 °C, 75% RH, and 1000–2000 ppm CO_2_ for 21 days. After spawn run, in order to satisfy the different fruiting requirement of different species, the conditions were adjusted to 21 ± 1°C, 90% RH, 150 lux light, and 500–600 ppm CO_2_ for *P.* cf. *floridanus* and *P. pulmonarius* strains in room 1 and 18 ± 1°C, 90% RH, 150 lux light, and 500–600 ppm CO_2_ for *P. ostreatus* strains in room 2, respectively [34,51,52]. The same substrate ingredients, formula, and incubation rooms were used for the two trials.

### 4.2. Bacteria Strain 

The bacterial strain (Pt011W) used in this study was isolated from brown blotch infected caps of cultivated *P. ostreatus* at the mushroom farm of Huazhong Agriculture University, China, 2017. The strain Pt011W, which has never been used for resistance screening before, was maintained at −80 °C in the ERCEMF. The identity of Pt011W was verified by 16S ribosomal RNA (16S rRNA), RNA polymerase beta subunit (rpoβ), and tolaasin biosynthesis gene. The total bacterial genomic DNA was isolated using a BioFlux Kit (Bioer Tech. Co. Ltd. China). PCR amplifications were performed with primer pairs 27F/1492R for 16S rRNA and rpoB-PSF/rpoB-PTR for rpoβ in a Bio-Rad T100 thermal cycler (Bio-Rad Lab. Inc. Ltd., USA) [53,54] and Pt-1A/ Pt-1D1 for tolaasin biosynthesis gene (Supplementary Table S2) [36]. PCR products were sequenced at Sangon Biotech Co. Ltd. Changchun, China. The resultant sequences of Pt011W were compared with sequences of *Pseudomonas* spp. in GenBank for homology using the BLASTn suite of the National Center for Biotechnology Information (NCBI). To infer the phylogenetic relationship of Pt011W within representative *Pseudomonas* spp., a neighbor-joining (NJ) tree from their concatenated 16S rRNA and rpoβ sequence alignment was constructed with a T92+G model and 1000 bootstrap replicates in MEGA 7 software (Supplementary Table S3) [55]. 

### 4.3. Inoculum Preparation

The single colony of *P. tolaasii* Pt011W was cultured in LB liquid medium (Solarbio S&T Co. Ltd., Beijing, China) and incubated at 25 °C for 16 h by orbital shaking at 150 rpm. After being centrifuged at 5000 rpm, the bacterial pellets were re-suspended in sterile distilled water. The density was adjusted to 0.3 absorbance at 450 nm using a spectrophotometer (Spectramax i3, Molecular Devices, LLC. San Jose, CA. USA) according to Rama et al. [39]. Viable cell count expressed as colony-forming units/mL (CFU/mL) was estimated by the spread plate method on LB solid medium at appropriate dilutions and incubated at 25 °C for 48 h. Three replicate spread plates were used to estimate mean cell count. The bacteria inoculum suspension provided ≈ 1.3 ± 0.2 × 10^8^ CFU/ml for both experiments.

### 4.4. Pathogenicity and the Efficacy of the IMP Method for Resistance Evaluation

The pathogenicity of *P. tolaasii* strain Pt011W and the efficacy of the IMP method for resistance evaluation was first verified using different concentrations of the bacterial suspension applied to three strains, P0 (*P. ostreatus*), P282 (*P.* cf. *floridanus*), and PPU (*P. pulmonarius*). Six dosages, 1.3 × 10^8^, 8.7 × 10^7^, 6.5 × 10^7^, 4.3 × 10^7^, 2.6 × 10^7^, 1.3 × 10^7^ CFU/mL, were prepared by diluting the initial inoculum (≈ 1.3 × 10^8^ CFU/mL) with sterile distilled water in the ratio 1:0, 1:0.5, 1:1, 1:2, 1:4, 1:9. The inoculation procedure involved dripping 20 µL aliquot of inoculum suspension onto the pileus surface (IMP) in situ was used [27]. When the pileus diameter was about 2–4 cm, the inoculum was dripped onto the pileus surface of three randomly selected caps/bag. For each strain, the treatment was performed on six bags as replications. Caps treated with sterile water served as controls. In the first experiment, all the fruiting bodies of mushroom in situ were incubated in room 1. The same experiment was repeated in room 2 (second experiment) using different bags but the same strains (P0, P282, and PPU) to detect whether the fruiting temperature had an influence on BBD severity.

Blotch symptoms appeared on the treated caps 24 hours after inoculation but failed to spread, except the portion inoculated on the pileus surface. The DS of each treated cap was assessed based on the blotch lesion morphology or the extent of discoloration [27]. The scale ratings were measured from 0–5 with 0 = no symptom; 0.5 = very pale lesion difficult to distinguish from the cap; 1 = level yellowish lesion; 2 = level brown lesions; 3 = slightly sunken lesion; 4 = extensive brown sunken lesion; 5 = extensive dark sunken lesions occasionally with sticky cytosolic material. Observation and rating of developed symptoms were carried out at 24, 48, and 72 hours after inoculation (HAI). 

### 4.5. Characterization of 97 Pleurotus Strains for Resistance to BBD

Two screening methods, IMP and IMSS, were used to investigate the resistance response of each *Pleurotus* strain to *P. tolaasii* Pt011W infection in two separate experiments. In 2017, a total of 97 *Pleurotus* strains were tested by the IMP method using ≈1 × 10^8^ CFU/mL of Pt011W inoculum suspension. The infection assay was performed as described above with slight modifications. All of the *P.* cf. *floridanus* and *P. pulmonarius* strains were incubated in room 1, and those of *P. ostreatus* in room 2. For each strain, the first flush fruiting bodies of 10 bags (three randomly selected caps/bag) were treated with the bacterial inoculum. Blotch symptoms were assessed visually at 48 HAI using the 0–5 lesion morphology scale mentioned above. The experiment was repeated for the second flush fruiting. Then the average DS score (DS1) of the two flushes/bag was determined for each strain. The DS1 was used to rate the reaction of the host as HR (DS1 ≤ 0.5), MR (DS1 ≤ 1.5), MS (DS1 ≤ 2.5), S (DS1 ≤ 3.5), or HS (DS1 > 3.5).

In 2018, the IMSS screening method was used to assess the resistance of the same 97 *Pleurotus* mushroom strains to Pt011W infection [31]. After 21 days of spawn run, each spawned substrate bag was sprayed with 1 ml of bacterial inoculum suspension (≈1 × 10^8^ CFU/mL) using a mini hand sprayer. The bags were further incubated for five days before the incubation conditions were adjusted in each room for the formation of fruiting bodies. During the fruiting period, the mushrooms were observed daily for the presence and development of blotch symptoms. Blotch symptoms developed on the pinhead fruiting bodies but were more visible on primordial and pileus surface. At maturity (diameter of caps about 2–4 cm), the severity of the disease was assessed based on blotch lesion size and the proportion (%) of cap surface area covered by the blotches on a scale of 0–3, where 0 =no symptom; 1= slight symptom with few spotty blotches (0.1–1% area affected); 2 = moderate symptom with many spotty blotches (1–5% area affected); 3 = severe symptom with many spotty or large blotches (>5% area affected) (adapted from [31,41]). To avoid substrate contamination, the first flush fruiting bodies were carefully harvested and bags were incubated for the second flush fruiting. Similar symptoms were observed and assessed in the second flush fruiting. From these two flushes, the mean DS score (DS2) from the 10 replicate bags (3 caps assessed/flush/bag) was determined for each strain. Host reactions were rated on the basis of the DS2 obtained as HR (DS2 ≤ 0.5), MR (DS2 ≤ 1.0), MS (DS2 ≤ 1.5), S (DS2 ≤ 2.0) or HS (DS2 > 2.0).

### 4.6. Detection of P. tolaasii on Mushroom Caps and Quantification in Spawned Substrate Bags

During the IMP and IMSS screening trials, blotch symptomatic caps were picked frequently for bacterial re-isolation. Cut tissue segments containing blotches were macerated in 10 ml sterilized distilled water and evenly spread on *Pseudomonas* Agar for the detection of fluorescein (PAF agar) (Solarbio S&T Co. Ltd., Beijing, China). The plates were incubated at 25 °C for 48 h. Based on morphology, the colonies were identified as previously described [35] to fulfill Koch’s postulates. The identities of the colonies were further confirmed by DNA extraction and PCR methods using two sets of primers Pt-1A/Pt-1D1 (5’ATCCCTTCGGCGTTTACCTG3’/5’CAAAGTAACCCTGCTTCTGC3’) and Pt-PM/Pt- QM (5’TGCCTTACGCGCTGATTGGC3’/5’TGATCAAACTCCAGCAATAG3’) [36].

For the quantification of the bacteria in the substrate bags, 14 strains selected randomly were assessed. The spawn material, 20 g/strain was collected from five bags (4 g/bag) after the first flush harvest. Then, 10 g aliquot was suspended in 50 ml of sterile distilled water and agitated on an orbital shaker (150 rpm) at 25 °C for 10 min. Nine serial dilutions with sterile water were prepared from the supernatant and 100 µL aliquots from each diluted suspension plated on PAF agar. Three replicates were prepared and subsequently incubated at 25 °C for 48 h. The colonies were identified, and their average count/strain determined by plate counting (CFU/mL), which was reported as CFU/10g of the spawned substrate. 

### 4.7. Statistical Analysis

Prior to each analysis, data were subjected to a Q–Q plot for normality and homogeneity of variance [56]. A 3-way crossed analysis of variance (ANOVA) model was used to determine the effects of temperature (21 °C and 18 °C), strain (P0, P282, and PPU), inoculum dosages (7 levels), and their interactions on BBD severity. The mixed procedure was used to estimate the variance components of each factor as a random effect by the Kenward and Roger approximation method [57]. The analysis was repeated for each disease assessment period (24, 48, and 72 HAI) to determine the best time for symptom evaluation. 

Data obtained from the two screening experiments and bacteria counts were subjected to ANOVA to detect statistical significance. Heterogeneous group means were separated with Duncan’s multiple range test (DMRT). For clarity in data presentation, the analysis of relative responses of mushroom strains was done for each species group. Rather than performing interspecies group contrast, the added variance due to species was estimated using the variance component analysis. Furthermore, a mixed effect model consisting of strains nested within species was used. 

Pearson correlation index (*r*) was used to examine the relationship between the behaviors of strains response to BBD in the two screening experiments, using their mean DS values as bivariate data. All statistical tests were performed at 5% significance level in Minitab^®^ 18.1 software (Minitab, LLC. USA) [58]. The average DS scores from the IMP screening method (DS1) and that from the IMSS method (DS2) were pooled for each strain tested to obtain DI, using the formula:

(1) $$\mathrm{DI}=0.5\;\left(\frac{1}{5}\mathrm{DS}1+\frac{1}{3}\mathrm{DS}2\right)\times \;100\;\%$$

where DI = overall disease severity index, DS = disease severity, and the denominators 5 and 3 represent the highest rating on the symptom severity scale of the IMP and IMSS method, respectively. The overall reaction of the host to BBD was rated based on DI as HR (DI ≤ 15.0%), MR (DI ≤ 25.0%), MS (DI ≤ 40.0%), S (DI ≤ 55.0%), or HS (DI > 55.1%).

## Figures and Tables

**Table 1:** Results of the ANOVA test and variance component estimate for the main, 2- and 3-way interactive effects of strain (S), incubation temperature (T), and inoculum dosage (D) on the progression of BBD severity, 24–72 hours after inoculation.

		Time (h) After the Inoculation of *P. tolaasii*					
		24 h	48 h	72 h	24 h	48 h	72 h
Source of variation	df	*p-*value	*p-*value	*p-*value	Variance component estimate (%)		
Strain	2	0.000	0.000	0.000	2.44	4.74	5.80
Temp	1	0.678	0.161	0.220	0.00	0.07	0.04
Dosage	6	0.000	0.000	0.000	86.01	82.15	81.99
Strain*Temp	2	0.863	0.789	0.970	0.00	0.00	0.00
Strain*Dosage	12	0.014	0.000	0.000	1.12	4.56	4.63
Temp*Dosage	6	0.911	0.751	0.757	0.00	0.00	0.00
Strain*Temp*Dosage	12	0.884	1.000	0.992	0.00	0.00	0.00
Error	210				10.44	8.47	7.55
Total	251				100.00	100.00	100.00

df = degrees of freedom; *p-*values < 0.05 were considered significant. * Interaction.

**Table 2:** BBD severity and resistance ratings of 56 *P. pulmonarius* strains based on screening with two methods via the inoculation of the pathogen on mushroom cap or pileus method (IMP) and on the mushroom spawned substrate method (IMSS).

					Screening with the IMP Method ^X^			Screening with the IMSS Method ^Y^			Overall Disease Response (DI) ^Z^	
No	Strain ID	Origin	Type	Cap Color	Disease Severity (DS1)		Resistance Rating	Disease Severity (DS2)		Resistance Rating	Overall DI (%)	Resistance Rating
1	F65	Yunnan, China	W	Brown	1.07 ± 0.21	a*	MR	1.13 ± 0.23	j–n*	MS	29.6	MS
2	F321	Yunnan, China	C	Brown	1.13 ± 0.17	a	MR	1.10 ± 0.35	j–m	MS	29.7	MS
3	F319	Yunnan, China	W	Dark brown	1.17 ± 0.28	a	MR	0.70 ± 0.25	f–h	MR	23.3	MR
4	F359	Heilongjiang, China	W	Pale brown	1.17 ± 0.24	a	MR	0.33 ± 0.42	b–e	HR	17.2	MR
5	F372	Yunnan, China	W	Pale brown	1.18 ± 0.25	a–b	MR	0.43 ± 0.42	c–f	HR	19.0	MR
6	F364	Yunnan, China	W	Pale brown	1.20 ± 0.23	a–b	MR	2.17 ± 0.33	t	HS	48.1	S
7	F10	Yunnan, China	W	Dark brown	1.23 ± 0.23	a–c	MR	0.30 ± 0.43	a–d	HR	17.3	MR
8	F13	Yunnan, China	W	Brown	1.23 ± 0.23	a–c	MR	1.83 ± 0.24	s	S	42.9	S
9	F7	Yunnan, China	W	Dark brown	1.23 ± 0.27	a–c	MR	2.40 ± 0.26	t	HS	52.3	S
10	F9	Yunnan, China	W	Brown	1.23 ± 0.27	a–c	MR	0.07 ± 0.21	a–b	HR	13.4	HR
11	F11	Yunnan, China	W	Brown	1.27 ± 0.31	a–d	MR	0.70 ± 0.19	f–h	MR	24.3	MR
12	F17	Jilin, China	W	Cream	1.27 ± 0.26	a–d	MR	0.97 ± 0.37	h–k	MR	28.8	MS
13	F333	Pakistan	W	Brown	1.27 ± 0.31	a–d	MR	0.27 ± 0.44	a–d	HR	17.1	MR
14	F368	Yunnan, China	W	Brown	1.27 ± 0.21	a–d	MR	1.63 ± 0.33	p–s	S	39.9	MS
15	F5	Yunnan, China	W	Cream	1.27 ± 0.21	a–d	MR	1.03 ± 0.25	i–l	MS	29.9	MS
16	F337	Pakistan	W	Pale brown	1.33 ± 0.35	a–e	MR	0.70 ± 0.33	f–h	MR	25.0	MR
17	F79	Heilongjiang, China	W	Pale brown	1.33 ± 0.35	a–e	MR	1.47 ± 0.17	o–r	MS	37.8	MS
18	F324	Yunnan, China	W	Cream	1.33 ± 0.22	a–e	MR	0.00 ± 0.00	a	HR	13.3	HR
19	F122	Yunnan, China	W	Pale brown	1.37 ± 0.39	a–e	MR	0.00 ± 0.00	a	HR	13.7	HR
20	F344	Heilongjiang, China	W	Pale brown	1.36 ± 0.48	a-e	MR	0.00 ± 0.00	a	HR	13.7	HR
21	F371	Yunnan, China	W	Pale brown	1.36 ± 0.36	a–e	MR	0.87 ± 0.17	h–j	MR	28.1	MS
22	F2	Yunnan, China	W	Pale brown	1.50 ± 0.36	b–f	MR	0.77 ± 0.39	g–i	MR	27.8	MS
23	F366	Yunnan, China	W	Brown	1.53 ± 0.23	c–f	MS	1.33 ± 0.42	l–p	MS	37.5	MS
24	F376	Yunnan, China	W	Pale brown	1.57 ± 0.32	d–f	MS	0.43 ± 0.42	c–f	HR	22.9	MR
25	F74	Jilin, China	W	Brown	1.60 ± 0.31	e–g	MS	1.57 ± 0.39	p–s	S	42.1	S
26	F365	Yunnan, China	W	Cream	1.63 ± 0.29	e–g	MS	0.83 ± 0.28	g–j	MR	30.2	MS
27	F6	Yunnan, China	W	Cream	1.70 ± 0.29	f–h	MS	0.67 ± 0.31	f–h	MR	28.1	MS
28	F21	Heilongjiang, China	W	Dark brown	1.77 ± 0.23	f–i	MS	1.70 ± 0.19	q–s	S	46.0	S
29	F363	Yunnan, China	W	Cream	1.77 ± 0.23	f–j	MS	2.20 ± 0.42	t	HS	54.3	S
30	F370	Yunnan, China	W	Brown	1.77 ± 0.27	f–i	MS	2.37 ± 0.19	t	HS	57.1	HS
31	F334	Pakistan	W	Cream	1.80 ± 0.23	f–k	MS	1.43 ± 0.32	n–q	MS	41.9	S
32	F318	Yunnan, China	W	Pale brown	1.90 ± 0.32	g–k	MS	1.77 ± 0.32	r–s	S	48.5	S
33	F14	Yunnan, China	W	Pale brown	1.97 ± 0.25	h–l	MS	0.63 ± 0.33	e–h	MR	30.2	MS
34	PPU	Jilin, China	C	Dark brown	2.07 ± 0.41	i–m	MS	1.50 ± 0.42	o–r	MS	45.7	S
35	F73	Jilin, China	W	Pale brown	2.23 ± 0.32	l–n	MS	1.50 ± 0.39	o–r	MS	47.3	S
36	F320	Yunnan, China	C	Brown	2.27 ± 0.22	m–o	MS	1.37 ± 0.48	m-p	MS	45.5	S
37	F20	Heilongjiang, China	W	Cream	2.30 ± 0.25	m–o	MS	2.17 ± 0.18	t	HS	59.1	HS
38	F3	Yunnan, China	W	Brown	2.33 ± 0.35	m–o	MS	1.43 ± 0.57	n–q	MS	47.2	S
39	F367	Yunnan, China	W	Brown	2.37 ± 0.37	m–o	MS	1.10 ± 0.23	j–m	MS	42.0	S
40	F96	Yunnan, China	W	Cream	2.47 ± 0.39	n–p	MS	0.93 ± 0.26	h–k	MR	40.2	S
41	F53	Sichuan, China	W	Brown	2.57 ± 0.32	o–q	S	2.87 ± 0.17	u	HS	73.4	HS
42	F369	Yunnan, China	W	Pale brown	2.67 ± 0.31	p–r	S	2.73 ± 0.44	u	HS	72.2	HS
43	F362	Yunnan, China	W	Brown	2.67 ± 0.38	p–r	S	1.23 ± 0.23	k–o	MS	47.2	S
44	F111	Jilin, China	W	Pale brown	2.70 ± 0.29	p–r	S	0.23 ± 0.42	a–d	HR	30.9	MS
45	F8	Yunnan, China	W	Pale brown	2.70 ± 0.33	p–r	S	0.87 ± 0.17	g–j	MR	41.4	S
46	F336	Pakistan	W	Brown	2.73 ± 0.26	p–s	S	0.83 ± 0.28	g–j	MR	41.2	S
47	F108	Yunnan, China	W	Pale brown	2.77 ± 0.23	p–s	S	0.20 ± 0.28	a–c	HR	31.0	MS
48	F86	Heilongjiang, China	W	Pale brown	2.83 ± 0.24	q–t	S	2.93 ± 0.26	u	HS	77.2	HS
49	F361	Yunnan, China	W	Brown	2.87 ± 0.42	q–t	S	0.53 ± 0.45	d–g	MR	37.6	MS
50	F81	Heilongjiang, China	W	Pale brown	2.97 ± 0.37	r–u	S	0.83 ± 0.18	g–j	MR	43.6	S
51	F68	Sichuan, China	W	Cream	3.03 ± 0.33	s–v	S	1.07 ± 0.31	i–m	MS	48.1	S
52	F1	Yunnan, China	W	Cream	3.10 ± 0.23	t–v	S	1.50 ± 0.24	o–r	MS	56.0	HS
53	F4	Yunnan, China	W	Brown	3.10 ± 0.41	t–v	S	1.57 ± 0.25	p–s	S	57.1	HS
54	F72	Jilin, China	W	Brown	3.10 ± 0.42	t–v	S	2.17 ± 0.18	t	HS	67.1	HS
55	F360	Yunnan, China	W	Brown	3.23 ± 0.32	u–v	S	2.20 ± 0.23	t	HS	69.0	HS
56	F18	Jilin, China	W	White	3.30 ± 0.25	v	S	0.63 ± 0.40	e–h	MR	43.6	S

^X^ The *P. pulmonarius* and *P.* cf. *florida* strains were cultivated in room 1 (at 21 ± 1 °C, 90% RH, 150 lux light, 500–600 ppm CO_2_) in 2017. Strain collection type: Wild (W) and cultivated (C).

DS1 = mean DS scores from two flushes produced/strain obtained after 48 hours of inoculation on mushroom caps/pilei of 10 replicate bags (3 caps were inoculated/flush/bag) ± standard deviation. Resistance scale based on DS1: 0–0.5 = highly resistant (HR); 0.6–1.5 = moderately resistant (MR); 1.6–2.5 = moderately susceptible (MS); 2.6–3.5 = susceptible (S); >3.5 = highly susceptible (HS).

^Y^ The *P. pulmonarius* and *P.* cf. *florida* strains were cultivated in room 1 (at 21 ± 1 °C, 90% RH, 150 lux light, 500–600 ppm CO_2_) in 2018. DS2 = mean DS scores from two flushes produced/strain, obtained during mushroom fruiting after inoculation of *P. tolaasii* on the spawned substrate, calculated from 10 replicate bags (3 caps scored/flush/bag) ± standard deviation. Resistance scale based on DS2: 0–0.5 = HR; 0.6–1.0 = MR; 1.1–1.5 = MS; 1.6–2.0 = S; >2.0 = HS.

^Z^ Overall resistance scale base on DI: 0.01–15.0% = HR; 15.1–25.0% = MR; 25.1–40.0% = MS; 40.1–55.0% = S; >55.1% = HS. DI, disease index.

* Means having a different letter(s) in the same column differed significantly (*p* < 0.05) according to the DMRT.

**Table 3:** BBD severity and resistance ratings of 21 *P.* cf. *florida* strains based on screening with two methods via the inoculation of the pathogen on mushroom cap or pileus method (IMP) and on the mushroom spawned substrate method (IMSS).

					Screening with the IMP Method ^X^			Screening with the IMSS Method ^Y^			Overall Disease Response (DI) ^Z^	
No	Strain ID	Origin	Type	Cap Color	Disease Severity (DS1)		Resistance Rating	Disease Severity (DS2)		Resistance Rating	Overall DI (%)	Resistance Rating
1	P290	Kunming, China	C	Cream	1.30 ± 0.25	a	MR	1.30 ± 0.29	g–h	MS	34.7	MS
2	P287	Kunming, China	C	Cream	1.43 ± 0.27	a–b	MR	0.00 ± 0.00	a	HR	14.3	HR
3	P301	Kunming, China	C	Cream	1.47 ± 0.28	a–b	MR	1.13 ± 0.28	f–g	MS	33.6	MS
4	P328	Pakistan	C	Cream	1.50 ± 0.24	a–b	MR	0.97 ± 0.33	e–f	MR	31.1	MS
5	P202	Shandong, China	W	Cream	1.57 ± 0.32	a–b	MS	1.80 ± 0.36	i–j	S	45.7	S
6	P289	Kunming, China	C	White	1.73 ± 0.31	b	MS	0.00 ± 0.00	a	HR	17.3	MR
7	P334	USA	W	Cream	1.73 ± 0.31	b	MS	0.00 ± 0.00	a	HR	17.3	MR
8	P298	Kunming, China	C	Cream	1.77 ± 0.47	b	MS	0.43 ± 0.27	b–c	HR	24.9	MR
9	P299	Kunming, China	C	White	2.13 ± 0.47	c	MS	2.37 ± 0.43	l–m	HS	60.8	HS
10	P326	Pakistan	W	White	2.13 ± 0.36	c	MS	1.57 ± 0.23	h–i	S	47.4	S
11	P296	Kunming, China	C	Cream	2.20 ± 0.28	c	MS	2.20 ± 0.39	k–l	HS	58.7	HS
12	P271	Kunming, China	C	Dark gray	2.33 ± 0.25	c	MS	2.53 ± 0.32	m	HS	65.2	HS
13	P332	Pakistan	W	Cream	2.63 ± 0.33	d	S	0.20 ± 0.42	a–b	HR	29.7	MS
14	P331	Pakistan	W	White	2.70 ± 0.43	d	S	0.80 ± 0.32	d–e	MR	40.3	S
15	P335	USA	W	White	2.70 ± 0.37	d	S	0.00 ± 0.00	a	HR	27.0	MS
16	P282	Yunnan, China	C	Pale gray	2.73 ± 0.41	d–e	S	2.87 ± 0.50	n	HS	75.1	HS
17	P309	Kunming, China	C	Cream	2.83 ± 0.32	d–f	S	2.27 ± 0.34	l–m	HS	66.1	HS
18	P316	Kunming, China	C	White	2.83 ± 0.39	d–f	S	2.13 ± 0.32	k–l	HS	63.9	HS
19	P329	Pakistan	W	Cream	2.93 ± 0.34	d–f	S	0.80 ± 0.28	d–e	MR	42.7	S
20	P325	Pakistan	W	White	3.07 ± 0.41	e–f	S	1.97 ± 0.33	j–k	S	63.5	HS
21	P330	Pakistan	W	Cream	3.10 ± 0.27	f	S	0.53 ± 0.32	c–d	MR	39.9	MS

See Table 2 for footnotes.

**Table 4:** BBD severity and resistance ratings of 20 *P. ostreatus* strains based on screening with two methods via the inoculation of the pathogen on mushroom cap or pileus method (IMP) and on the mushroom spawned substrate method (IMSS).

					Screening with the IMP method ^X^			Screening with the IMSS method ^Y^			Overall disease response (DI) ^Z^	
No	Strain ID	Origin	Type	Cap Color	Disease Severity (DS1)		Resistance Rating	Disease Severity (DS2)		Resistance Rating	Overall DI (%)	Resistance Rating
1	P10	Changchun, China	C	Gray	1.53 ± 0.39	a*	MS	2.63 ± 0.48	h–i*	HS	59.2	HS
2	P164	Heilongjiang, China	C	Gray	1.57 ± 0.27	a	MS	1.80 ± 0.45	d–f	S	45.7	S
3	P92	Changchun, China	W	Gray	1.77 ± 0.23	a	MS	1.17 ± 0.28	c	MS	37.1	MS
4	P191	Shandong, China	C	Pale gray	2.23 ± 0.42	b	MS	2.20 ± 0.32	g	HS	59.0	HS
5	P179	Heilongjiang, China	C	Gray	2.27 ± 0.26	b	MS	2.33 ± 0.44	g–h	HS	61.6	HS
6	P297	Yunnan, China	C	Pale gray	2.43 ± 0.39	b	MS	2.23 ± 0.50	g	HS	61.5	HS
7	P35	Yunnan, China	W	Dark gray	2.53 ± 0.48	b–c	S	1.60 ± 0.38	d	S	52.0	S
8	P11	Changchun, China	C	Pale gray	2.60 ± 0.41	b–c	S	2.00 ± 0.42	e–g	S	59.3	HS
9	P90	Changchun, China	W	Pale gray	2.60 ± 0.31	b–c	S	1.70 ± 0.37	d–e	S	54.3	S
10	P67	Changchun, China	W	Dark gray	2.87 ± 0.28	c–d	S	2.96 ± 0.20	i	HS	78.0	HS
11	P176	Heilongjiang, China	C	Gray	3.00 ± 0.39	d	S	0.97 ± 0.25	b–c	MR	46.1	S
12	P186	Heilongjiang, China	C	Dark gray	3.10 ± 0.49	d–e	S	2.93 ± 0.44	i	HS	79.9	HS
13	P279	Yunnan, China	C	Pale gray	3.20 ± 0.39	d–f	S	2.30 ± 0.37	g–h	HS	70.3	HS
14	P317	Yunnan, China	C	Pale gray	3.20 ± 0.36	d–g	S	0.70 ± 0.25	b	MR	43.7	S
15	P346	Yunnan, China	C	Cream	3.23 ± 0.42	d–h	S	0.00 ± 0.00	a	HR	32.3	MS
16	P62	Yunnan, China	W	Dark gray	3.43 ± 0.35	e–h	S	1.83 ± 0.33	d–f	S	64.9	HS
17	P173	Heilongjiang, China	C	Dark gray	3.57 ± 0.32	f–i	HS	2.63 ± 0.40	h–i	HS	79.6	HS
18	P40	Yunnan, China	C	Dark gray	3.88 ± 0.42	i	HS	2.70 ± 0.48	i	HS	83.7	HS
19	P0	Jilin, China	C	Pale gray	3.90 ± 0.32	i	HS	2.10 ± 0.39	f–g	HS	74.0	HS
20	P167	Heilongjiang, China	C	Dark gray	4.33 ± 0.52	j	HS	1.70 ± 0.29	d–e	S	71.7	HS

^X^ The *P. ostreatus* strains were cultivated in room 2 (at 18 ± 1 °C, 90% RH, 150 lux light, 500–600 ppm CO_2_) in 2017. Strain collection type: Wild (W) and cultivated (C).

DS1 = mean DS scores from two flushes produced/strain obtained after 48 hours of inoculation on mushroom caps/pilei of 10 replicate bags (3 caps were inoculated/flush/bag) ± standard deviation. Resistance scale based on DS1: 0–0.5 = highly resistant (HR); 0.6–1.5 = moderately resistant (MR); 1.6–2.5 = moderately susceptibly (MS); 2.6–3.5 = susceptible (S); >3.5 = highly susceptible (HS).

^Y^ The *P. ostreatus* strains were cultivated in room 2 (at 18 ± 1 °C, 90% RH, 150 lux light, 500–600 ppm CO_2_) in 2018. DS2 = mean DS scores from two flushes produced/strain, obtained during mushroom fruiting after inoculation of *P. tolaasii* on the spawned substrate, calculated from 10 replicate bags (3 caps scored/flush/bag) ± standard deviation. Resistance scale based on DS2: 0-0.5 = HR; 0.6-1.0 = MR; 1.1-1.5 = MS; 1.6-2.0 = S; >2.0 = HS.

^Z^ Overall resistance scale base on DI: 0.01–15.0% = HR; 15.1–25.0% = MR; 25.1–40.0% = MS; 40.1–55.0% = S; >55.1% = HS.

* Means having a different letter(s) in the same column differed significantly (*p* < 0.05) according to the DMRT.

**Table 5:** Variance estimate attributed to species and strains of *Pleurotus* in resistance to *P. tolaasii,* after screening with the IMP method and the IMSS method.

Source of Variation	Degree of Freedom	Sum of Squares	Mean Squares	*F*-value	*p*-value	Variance Component	Percent of Total
	Screening by the IMP method						
Species	2	122.98	61.49	12.58	0.000	0.20	25.44
Strain (Species)	94	459.55	4.89	45.03	0.000	0.48	60.76
Error	873	94.78	0.11			0.11	13.80
Total	969	677.31				0.79	100.00
	Screening by the IMSS method						
Species	2	82.09	41.04	6.41	0.002	0.13	14.96
Strain (Species)	94	601.89	6.40	58.02	0.000	0.63	72.35
Error	873	96.34	0.11			0.11	12.69
Total	969	780.32				0.87	100.00

The variance component was estimated with a mixed effect nested ANOVA. *p-*values < 0.05 were significant.

**Table 6:** The number of *Pleurotus* strains and their resistance classes according to their overall disease index (DI).

Resistance Class^*^	*P. pulmonarius*	*P. ostreatus*	*P.* cf. *florida*	Total
HR (1.0–15.0%)	4	0	1	5
MR (15.1–25.0%)	8	0	3	11
MS (25.1–40.0%)	15	2	6	23
S (40.1–55.0%)	20	5	4	29
HS (55.1–80.0%)	9	13	7	29
Total	56	20	21	97

^*^ Resistance classes are based on DI ranges (in %) obtained by combining the mean DS obtained in both screening methods (IMP and IMSS) for each strain evaluated. Highly resistant (HR); moderately resistant (MR); moderately susceptible (MS); susceptible (S); highly susceptible (HS).

**Table 7:** Quantitative recovery of *P. tolaasii* from 10 g spawn substrate among strains of three *Pleurotus* species after harvesting the first flush of fruiting bodies.

*P. pulmonarius*	No. of *P. tolaasii* CFU (× 10^7^)	*P. ostreatus*	No. of *P. tolaasii* CFU (× 10^7^)	*P.* cf. *florida*	No. of *P. tolaasii* CFU (× 10^7^)
F359	27.8 ± 12.4 a	P176	32.8 ± 11.6 a	P334	17.3 ± 12.5 a
F111	30.5 ± 11.4 a	P191	45.3 ± 12.7 a	P301	30.0 ± 10.8 a–b
F369	47.0 ± 11.1 a–b	P0	55.5 ± 11.3 a	P271	43.3 ± 11.3 b–c
PPU	53.5 ± 10.0 b–c	P186	63.7 ± 11.9 a	P282	62.5 ± 14.2 c
F53	69.0 ± 10.2 c				
F86	75.0 ± 13.4 c				

Means with a different letter(s) in the same column differed significantly (*p* < 0.05) according to the DMRT test. Data were square-root transformed prior to ANOVA; the reported are untransformed means. Viable counts were estimated from three replicate spread-plates and expressed as CFU/10 g substrate for each strain sampled ± standard deviation.

## Supplementary Materials

The following are available online at https://www.mdpi.com/2076-0817/8/4/227/s1. Figure S1. The phylogenetic relationship between *P. tolaasii* strain Pt011W and other *Pseudomonas* spp., inferred by the neighbor-joining method based on the concatenated 16S rRNA and rpoβ gene sequence data; Table S1. List of fungal strains used in the study, herbarium ID, and site of collection; Table S2. List of genes and primers used for characterization of *P. tolaasii* strain Pt011W; Table S3. *Pseudomonas* specie*s* and GenBank accession numbers of strains used in phylogenetic analyses.

## Abbreviations

BBD: Brown blotch disease; DI: Disease index; DS: Disease severity; HAI: Hours after inoculation; IMP: Inoculation of the pathogen on mushroom pileus; IMSS: Inoculation of the pathogen on mushroom spawned substrate; HR: Highly resistant; MR: Moderately resistant; MS: Moderately susceptible; S: Susceptible; HS: Highly susceptible.
