# Supplementary material for: Resistance Sources to Brown Blotch Disease (Pseudomonas tolaasii) in a Diverse Collection of Pleurotus Mushroom Strains
Source: Pathogens. 2019 Nov 9;8(4):227. doi: 10.3390/pathogens8040227 (PMC6963638; doi:10.3390/pathogens8040227)
Supplement: Supplementary file 1 [file pathogens-08-00227-s001.zip › Supplementary Tables S2-S3.docx]

**Supplementary Tables S2-S3**

**Table S2.** List of genes and primers used for characterization of *P. tolaasii* strain Pt011W.

| **Gene** | **Name of Primer** | **Sequences**  **5' -3'** | **Annealing temperature/Reference** |
| --- | --- | --- | --- |
| 16S rRNA | 27F | AGAGTTTGATCMTGGCTCAG | 60^o^C  Godfrey et al., 2010 |
|  | 1492R | TACGGYTACCTTGTTACGACTT |  |
| rpoβ | rpoB-PSF | AGTTCATGGACCAGAACAACC | 55 ^o^C  Sajben et al., 2011 |
|  | rpoB-PTR | CCTTGACGGTGAACTCGTTC |  |
| Tolaasin | Pt-1A | ATCCCTTCGGCGTTTACCTG | 58^o^C  Lee et al., 2002 |
|  | Pt-1D1 | CAAAGTAACCCTGCTTCTGC |  |
|  | Pt-PM | TGCCTTACGCGCTGATTGGC | 58^o^C |
|  | Pt-QM | TGATCAAACTCCAGCAATAG |  |

**Table S3.** *Pseudomonas* specie*s* and GenBank accession numbers of strains used in phylogenetic analyses.

| Species | Strain | GenBank Accession No. | |
| --- | --- | --- | --- |
|  |  | 16S rRNA | rpoβ |
| *P. tolaasii* | Pt011W | MN630174 | MN630175 |
| *P. tolaasii* | NCPPB 2192 | NR_114595.1 | CP020369.1 |
| *P. tolaasii* | LMG 2342 | NR_041799.1 | AJ717467.1 |
| *P. palleroniana* | CFBP 4389 | NR_029050.1 | NA |
| *P. costantinii* | CFBP 5705 | NR_025164.1 | NA |
| *P. lurida* | P 513/18 | NR_042199.1 | HE800513.1 |
| *P. reactans* | LMG 5329 | AF255337.1 | HM070042.1 |
| *P. agarici* | LMG 2112T | Z76652.1 | AJ717477.1 |
| *P. fluorescens* | NCTC10038 | LS483372.1 | LS483372.1 |
