# Supplementary material for: Resistance Sources to Brown Blotch Disease (Pseudomonas tolaasii) in a Diverse Collection of Pleurotus Mushroom Strains
Source: Pathogens. 2019 Nov 9;8(4):227. doi: 10.3390/pathogens8040227 (PMC6963638; doi:10.3390/pathogens8040227)
Supplement: Supplementary file 1 [file pathogens-08-00227-s001.zip › Supplementary Figure S1.docx]

**Supplementary Figure S1**


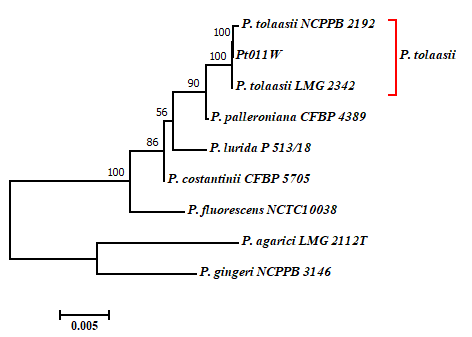


**Figure S1.** The phylogenetic relationship between *P. tolaasii* strain Pt011W and other *Pseudomonas* spp., inferred by the Neighbor-Joining method based on the concatenated 16S rRNA and rpoβ gene sequence data. The tree is rooted with *P. agarici* and *P. gingeri*. Clade support values indicated (in %) on branches represent the proportions of confidence determined by 1000 bootstrap replicate tests.
